# Factors affecting voluntary HIV counselling and testing among men in Ethiopia: a cross-sectional survey

**DOI:** 10.1186/1471-2458-12-438

**Published:** 2012-06-15

**Authors:** Tesfaye H Leta, Ingvild F Sandøy, Knut Fylkesnes

**Affiliations:** 1 Centre for International Health, University of Bergen, Overlege Danielsens Hus, Årstadv. 21, Postbox 7804, NO-5020 Bergen, Norway; 2 Orthopaedic Clinic, Haukeland University Hospital, Jonas Lies vei 65, 5021 Bergen, Norway

**Keywords:** HIV testing, VCT utilization, Stigma, Knowledge, Men, Ethiopia

## Abstract

**Background:**

Voluntary HIV counselling and testing (VCT) is one of the key strategies in the HIV/AIDS prevention and control programmes in Ethiopia. However, utilization of this service among adults is very low. The aim of the present study was to investigate factors associated with VCT utilization among adult men since men are less likely than women to be offered and accept routine HIV testing.

**Methods:**

The study utilized data from the Ethiopian Demographic Health Survey (EDHS) 2005, which is a cross-sectional survey conducted on a nationally representative sample. Using cluster sampling, 6,778 men aged 15–59 years were selected from all the eleven administrative regions in Ethiopia. Logistic regression was used to analyze potential factors associated with VCT utilization.

**Results:**

Overall, 21.9% of urban men and 2.6% of rural men had ever tested for HIV through VCT and most of them had learned their HIV test result. Having no stigmatizing attitudes toward people living with HIV/AIDS was found to be strongly and positively associated with VCT utilization in both urban and rural strata. In rural areas HIV test rates were higher among younger men (aged ≤44 years) and those of higher socio-economic position (SEP). Among urban men, risky sexual behaviour was positively associated with VCT utilization whereas being Muslim was found to be inversely associated with utilization of VCT. Area of residence as well as SEP strongly affected men’s level of stigmatizing attitudes toward people living with HIV/AIDS.

**Conclusions:**

VCT utilization among men in Ethiopia was low and affected by HIV/AIDS-related stigma and residence. In order to increase VCT acceptability, HIV/AIDS prevention and control programs in the country should focus on reducing HIV/AIDS-related stigma. Targeting rural men with low SEP should be given first priority when designing, expanding, and implementing VCT services in the country.

## Background

Ethiopia is one of the countries in sub-Saharan Africa (SSA) that have been affected by a generalised HIV/AIDS epidemic [1]. The estimated adult HIV prevalence in 2009 was between 1.4% and 2.8% [2]. Even though this prevalence is lower than in many other SSA countries, Ethiopia has one of the largest populations of HIV-infected people in the world, with an estimate of 1.1million [3].

To combat the epidemic, the Ethiopian government has responded favourably ever since the first case of HIV was recorded in the country, using diverse approaches to mitigate the increasing and devastating effect of the HIV/AIDS epidemic [4,5]. The country started VCT for the larger community after the national HIV/AIDS policy was launched in 1998 [1], aiming to create an enabling environment to fight the pandemic. Over the years, the country has given increased support and commitment to developing specific HIV/AIDS-related legislation and revising the HIV policy to promote and protect human rights. In addition, the involvement of civil society in the process of planning, monitoring and evaluation of HIV/AIDS responses at various levels is improving [2].

VCT may have potential preventive effects on HIV transmission and serves as a gateway to most HIV/AIDS-related services [6,7]. The counselling component should be based on confidentiality and include information about HIV transmission and personal discussion about an individual’s risk in order to enable people to make informed decisions about testing and their own risk. Thus, expanding access to VCT services has both individual and societal benefits [8]. For the individual, VCT enhances the ability to reduce one’s risk of acquiring or transmitting HIV, to access HIV-specific treatment, care and support [3,5], to manage one’s health, and to plan for the future [8]. VCT is also vital for providing access to emotional support, improving skills to cope with HIV-related anxiety, and increasing motivation to avoid risky behaviours [9]. Furthermore, counselling and testing provide awareness of safer options in preventing vertical HIV transmission if pregnant women and their families use such services and learn about their sero-status [6,8,9]. For society, widespread knowledge of one’s HIV status can lead to better community mobilization against the epidemic, and may reduce HIV-related stigma and discrimination [8] and support human rights [6].

Despite the potential benefits of VCT, uptake is often poor regardless of the availability of the services [6,8,10-14]. Several possible contributing factors could play an essential role in the uptake of VCT: socio-demographic characteristics, proximity to a clinic [10-12], awareness/knowledge related to HIV/AIDS [11,12,15], perception of being at risk of HIV infection [12,15,16], perceived benefits of VCT [6,17], the belief that knowledge of infection may accelerate disease progression [9], psychosocial factors such as HIV/AIDS-related stigma and discrimination [8,10,11,16], and concerns about confidentiality [10,11,16].

Despite the various efforts made to implement HIV prevention activities [18], VCT uptake among adults has also been disappointingly low in Ethiopia. According to the 2005 Ethiopia Demographic Health Survey [EDHS] only 4% of women (aged 15–49 years) and 6% of men (aged 15–49 years) had ever been tested for HIV [14]. The Ethiopian government has therefore recently started routine HIV testing as well as integration of HIV counselling and testing with family planning and maternal, newborn and child health services [2]. Studies from other SSA countries indicate that the introduction of routine testing has particularly increased testing experience among women through PMTCT programs [19,20],whereas men are reluctant to come to the antenatal clinic with their wives to be tested [21-23]. It thus seems that improvement of voluntary counselling and testing services may be needed to increase uptake of testing among men. VCT places more emphasis on autonomy than routine testing, and is therefore better option from a human rights perspective too.

Men in Ethiopia, as in many other African countries, are the key decision-makers at home, in workplaces, in parliament and in religious institutions. In addition, men control economic resources that might be significant for HIV prevention and care. Since women in most societies in Africa need the consent of their partner to go to VCT, improving men’s utilization of VCT may directly or indirectly encourage women’s VCT utilization [24]. Only a few studies [25-28] related to voluntary HIV counselling and testing have been conducted in Ethiopia. It is against this background that the present study aims to investigate factors affecting voluntary counselling and testing of HIV among men aged 15–59 years in Ethiopia.

## Methods

### Design and study area

This study was based on data from the 2005 EDHS; the most recent national dataset on HIV testing that is available (as of January 2012). The 2005 EDHS included a nationally representative sample of women (aged 15–49 years) and men (aged 15–59 years) from all eleven administrative regions in the country [14]. This paper focused only on men aged 15–59 years. Although the testing rate in Ethiopia is likely to have increased since 2005 with the introduction of routine testing, this increase is probably more pronounced among women than men [19]. Thus, examining factors associated with VCT uptake among men in 2005 is still likely to yield information relevant for VCT programmes in the country.

### Study population and sampling procedure

**Figure 1:**
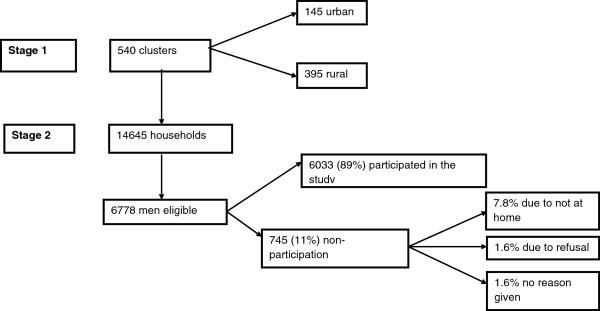
Sampling procedure and participation rate.

The 2005 EDHS used a non-proportionate two-stage sampling design with stratification into urban and rural areas. At stage one of the sampling, 540 clusters (145 urban and 395 rural) were selected on the basis of the 1994 Ethiopian population and housing census sample frame [14]. The second stage involved a complete listing of households in each selected cluster followed by a systematic random sampling of approximately 24–32 households from each cluster (giving a total of 14,645 households). In about half of all selected households, men aged 15–59 were eligible for interview [14] (Figure 1).

### Data collection

Eligible men who gave informed consent were interviewed by well-trained fieldworkers about their socio-demographic characteristics, sexual behaviour history, HIV/AIDS-related knowledge, and HIV/AIDS-related stigma.

### Measurements

The dependent variable for this study was VCT utilization. This was measured by asking the question: ‘Have you ever tested for HIV?’ The independent variables included socio-demographic characteristics: age, marital status, religion, residence, socio-economic position, HIV/AIDS-related knowledge, sexual behaviour, and HIV/AIDS-related stigma towards people living with HIV/AIDS.

### Operational definitions

An index of the study participants’ socio-economic position was developed on the basis of their educational level, wealth index, and occupational status. Primarily each sub-variable were grouped into three categories and then added together. This summation index gave a value ranging from 2 to 9. Participants’ SEP was categorized as low (score 2–5), middle (score 6–7), and high (score 8–9). Religion was categorized into two groups: Christian and Muslim. Respondents who listed religion as “other” were excluded from the analysis because the subgroup was small (n = 130). An HIV/AIDS-related knowledge index was built from the answers to seven questions: three questions on knowledge of HIV prevention and four on misconceptions about modes of HIV transmission. It was categorized as low (score ≤4), high (score 5–6), or comprehensive (score 7) knowledge. In order to assess their sexual behaviour history, participants were given eight questions related to their sexual behaviours (see Table 1) prior to the survey. These were combined into an index of risky sexual behaviour with three categories: “No risk” (score 0), “Some risk” (score 1) and “High risk” (score ≥2). Seven questions that reflected negative attitudes about people living with HIV/AIDS were used to create a stigma index. This index was categorized as “No stigma” (score 7), “Low stigma” (score 5–6), “Moderate stigma” (score 3–4), and “High stigma” (score ≤ 2).

**Table 1:** VCT uptake in relation to HIV/AIDS-related knowledge, HIV/AIDS-related stigma and risky sexual behaviour history among men in Ethiopia, 2005

**Variables**	**Urban**		**Rural**	
	**n (%)**	**Ever HIV tested (%)**	**n (%)**	**Ever HIV tested (%)**
**Overall**	**1,628 (27.0)**	**355 (21.9)**	**4,405 (73.0)**	**110 (2.6)**
**Knowledge indicator**				
**R**educe risk of getting AIDS by not having sex at all (yes)	1,460 (90.2)	22.3	3,362 (81.0)	2.9
**R**educe chance of getting AIDS by having one sexual partner with no other partner (yes)	1,429 (88.3)		3,263 (78.5)	2.9
**R**educe chances of getting HIV/AIDS by always using condoms during sexual intercourse (yes)	1,315 (81.3)	23.2	2,531 (60.9)	3.1
**C**an a healthy looking person have AIDS (yes)	1,450 (89.6)	23.7	2,684 (64.6)	3.4
**G**et AIDS by sharing food with person who has HIV/AIDS (no)	1,542 (95.3)	22.2	3,264 (78.7)	3.0
**G**et HIV/AIDS from mosquito bites (no)	1,289 (79.6)	22.7	2,268 (54.6)	3.3
**C**an get AIDS by witchcraft or supernatural means (no)	1,554 (96.0)	22.4	3,502 (84.3)	2.9
**Risky sexual behaviour indicators**				
**N**ever had sexual intercourse	525 (32.4)	13.5	1,203 (28.9)	1.6
**H**ad multiple life time sexual partner (yes)	746 (47.3)	27.5	1,591 (38.4)	3.1
**C**ircumcised (no)	31 (1.9)	6.5	360 (8.7)	1.9
**H**ad genital sore/ulcer in last 12 months (yes)	6 (0.4)	50.0	12 (0.3)	8.3
**H**ad any STD in last 12 months (yes)	8 (0.5)	37.5	23 (0.6)	4.4
**A**t least one sexual partner other than wife in last 12 months	272 (16.9)	36.0	183 (4.4)	3.3
**E**ither partner drank alcohol during last sex with last sexual intercourse (yes)	107 (12.7)	25.2	196 (7.2)	1.5
**E**ver paid for sex (yes)	20 (2.4)	35.0	22 (0.8)	0.0
**Stigma indicators**				
**K**nows someone who has or died of AIDS (yes)	640 (39.5)	31.3	271 (6.5)	9.2
**A**llowed to keep AIDS infection secret (no)	1,271 (78.5)	22.7	3,173 (76.3)	2.8
**W**illing to care for relative with AIDS (yes)	1,517 (93.9)	22.7	2,940 (70.7)	3.1
**P**erson with AIDS allowed to continue teaching (yes)	1,368 (84.5)	23.3	1,959 (47.1)	3.6
**W**ould buy vegetables from vendor with AIDS (yes)	1,079 (66.7)	25.7	877 (21.1)	4.6
**P**eople with AIDS should be ashamed of themselves (Disagree)	1,503 (92.8)	22.4	2,256 (54.2)	3.3
**P**eople with AIDS should be blamed for bringing AIDS in community (Disagree)	1,443 (89.1)	23.0	2,252 (52.2)	3.6

### Data analysis

**Figure 2:**
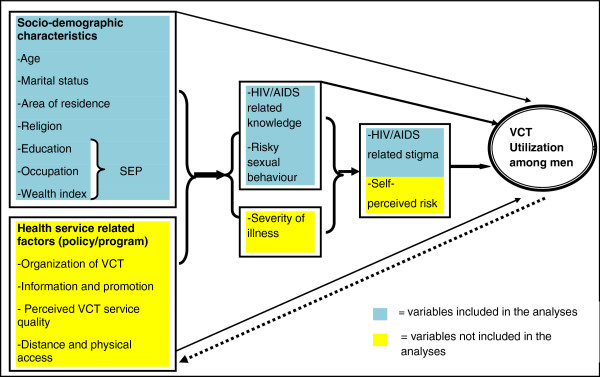
Suggested conceptual and analytical framework for studying determinants of VCT utilization by men and possible predicator variables.

The data were analyzed using STATA version 10. Sampling weights, cluster effects, and stratification of urban and rural areas were taken into consideration throughout the analyses. Logistic regression was used to assess the associations between the dependent and independent variables. In the regression models, information on age, marital status, SEP, religion, HIV/AIDS-related stigma, HIV/AIDS-related knowledge, and risky sexual behaviour history, were included as independent variables (see framework in Figure 2).

The selection of these variables was based upon our own hypotheses and previous findings from the literature [29]. Unfortunately, the EDHS 2005 did not collect information on all the factors that are likely to influence VCT use among men, but we have indicated some of these in the conceptual and analytical framework shown in yellow in Figure 2. First, collinearity among the independent variables was assessed by using a correlation matrix, and multicolinearity was not found. Secondly, the association between the dependent and each independent variable was analyzed separately in univariate analyses. Thereafter, a stepwise approach was employed in building the multivariate models. In the first model, the association between the socio-demographic variables and VCT was assessed. In the second model, both socio-demographic characteristics and more proximal determinants (HIV/AIDS-related knowledge and risky sexual behaviour) were included. In the final model, all variables were included. The associations were presented as odds ratios (OR) and 95% confidence intervals. The analyses were stratified by residence (urban and rural) due to interaction. In order to assess possible bias from non-participation on the findings, sensitivity analysis was performed under two different assumptions; i.e. HIV test prevalence ratios for non-participants vs. participants were assumed to be 1.5 and 2.

### Ethical consideration

Ethical approval for the EDHS 2005 was obtained from the National Ethics Review Committee of the Ethiopia Science and Technology Commission in Addis Ababa, Ethiopia and the Measure DHS, ORC Macro Institutional Review Board in Calverton, USA. The objective of the study was explained to eligible study participants. Informed consent was required and obtained from the participants before they were interviewed [14].

## Results

### Background characteristics of the study participants

A total of 6,033 men were enrolled in the study from the 6,778 who were eligible, yielding a response rate of 89% (Figure 1). The mean age of the participants was 31 (SD ± 11.7) years, with a median age of 29 years. The younger age group (15–24 years) represented about 38% of the study participants. The majority of the study participants were married and affiliated to Christianity (Table 2).

**Table 2:** Frequency distribution of VCT uptake among men in Ethiopia in 2005 by urban–rural residence

**Variables**	**Urban**			**Rural**		
	**n (%)**	**Ever HIV tested (%)**	***P*-value**	**n (%)**	**Ever HIV tested (%)**	***P*-value**
**Overall**	**1,628 (27.0)**	**355 (21.9)**		**4,405 (73.0)**	**110 (2.6)**	
**Age**						
15–24	688 (42.5)	18.8	**0.001**	1,514 (36.4)	2.6	**0.000**
25–34	437 (27.0)	27.9		1,102 (26.5)	4.4	
35–44	272 (16.8)	24.3		835 (20.1)	2.2	
45–59	222 (13.7)	17.1		709 (17.0)	0.6	
**Marital status**						
Never married	919 (56.8)	22.7	0.364	1,419 (34.1)	1.9	**0.032**
Ever Married	700 (43.2)	20.9		2,741 (65.9)	3.0	
**Socio-economic position (SEP)**						
Low	135 (8.3)	8.9	**0.000**	3,198 (76.9)	1.7	**0.000**
Middle	459 (28.4)	15.0		677 (16.3)	4.9	
High	1,025 (63.3)	26.7		285 (6.9)	8.1	
**Religion**						
Christian	1,215 (75.6)	24.4	**0.000**	2,544 (63.1)	2.7	0.969
Muslim	393 (24.4)	14.5		1,486 (36.9)	2.7	
**HIV/AIDS-related knowledge index**						
Low	134 (8.3)	16.7	**0.001**	1,381 (33.2)	1.1	**0.000**
High	621 (38.4)	19.0		1,860 (44.7)	3.1	
Comprehensive knowledge	864 (53.4)	25.4		919 (22.1)	4.1	
**HIV/AIDS-related stigma index**						
No stigma	335 (20.7)	33.4	**0.000**	46 (1.1)	15.2	**0.000**
Low	966 (59.8)	21.4		1,066 (25.6)	4.9	
Moderate	270 (16.7)	11.9		1,632 (39.2)	2.0	
High	48 (3.0)	8.3		1,416 (34.0)	1.3	
**Risky sexual behaviour index**						
No risk	543 (33.5)	14.2	**0.000**	1,082 (26.0)	1.8	0.104
Some risk	729 (45.0)	22.2		2,513 (60.4)	3.0	
High risk	347 (21.4)	33.4		565 (13.6)	2.8	

### HIV testing history

Fifty-three percent of the study participants reported that they knew a place where they could get tested for HIV, and 21.9% of urban men and 2.6% of rural men had ever tested for HIV. Men with the highest SEPs and men who were Christian were more likely to have ever been tested for HIV than those of lower SEP and those who were Muslim, respectively (Table 2). The overall prevalence of ever having tested for HIV among men was 8.1%. This prevalence became 8.5% and 10.3% in the first and second scenario, respectively, as we assessed sensitivity analysis.

### HIV/AIDS-related knowledge

A great proportion of the study participants (96%) reported that they had heard of HIV, of these, 90.2% of urban men and 80.3% of rural men responded that they knew how to prevent/avoid HIV infection. The level of knowledge was higher among urban than rural men on all measured parameters (Table 1).

### Risky sexual behaviour history

Of the total sample, about 47% of urban men and 38% of rural men reported two or more lifetime sexual partners. Twenty-eight percent of urban men and 3% of rural men, who reported multiple lifetime sexual partners, had ever tested for HIV. A higher proportion of urban than rural men had taken alcohol during the previous sexual intercourse (Table 1).

Overall, 66% of men in the urban stratum and 73% in the rural stratum who had ever had sexual intercourse reported one or more recent risky sexual behaviours. Twenty-six percent of urban men and 3% of rural men, who admitted one or more risky sexual behaviours, had ever tested for HIV.

### HIV/AIDS-related stigma

**Figure 3:**
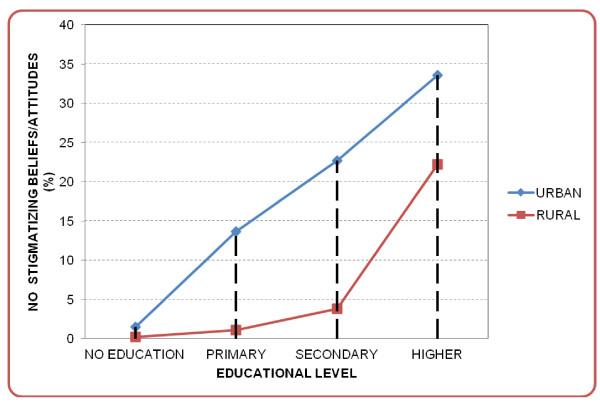
Study participants’ level of HIV/AIDS-related stigma versus level of education by area of residence.

Sixty percent of urban men and 93% of rural men reported not knowing anyone infected with HIV or who had died of AIDS. While 16% of urban men and 2% of rural men who reported not knowing anyone infected with HIV, or anyone who had died of AIDS, had ever tested for HIV, 31% of urban men and 9% of rural men who had had such acquaintances had been tested (Table 1). Generally, the majority had one or more stigmatizing beliefs towards people living with HIV/AIDSs (Table 2). While 33% of urban men and 15% of rural men with no stigmatizing attitudes had ever tested for HIV, 19% of urban men and 2.5% of rural men with stigmatizing beliefs towards people living with HIV/AIDS had been tested (Table 2 and Figure 3).

### Factors associated with VCT utilization

In the univariate analyses, utilization of VCT among men was found to be strongly and positively associated with SEP and strongly and negatively associated with stigma in both urban and rural strata. Reporting risky sexual behaviour was positively associated with VCT utilization in both strata, whereas being married and high or comprehensive HIV/AIDS-related knowledge were significantly and positively associated with VCT utilization only in the rural stratum. In urban areas, being a Muslim was negatively associated with VCT utilization (Tables 3 and 4).

**Table 3:** Logistic regression analysis of determinants of VCT utilization among urban men in Ethiopia, 2005

**Variables**	***Ever been tested for HIV***			
	**Univariate OR(95% CI)**♣	Model 1 OR(95% CI)*	Model 2 OR(95% CI)**	Full Model OR(95% CI)***
**Age**				
15–24	1.4 (0.76–2.52)	1.2 (0.56–2.70)	1.8 (0.88–3.61)	1.7 (0.78–3.59)
25–34	**1.9 (1.07–3.36)**	1.6 (0.87–2.86)	1.6 (0.91–2.73)	1.5 (0.81–2.66)
35–44	1.3 (0.73–2.33)	1.2 (0.66–2.09)	1.2 (0.66–2.07)	1.1 (0.60–2.07)
45–59	1	1	1	1
**Marital status**				
Never married	1	1	1	1
Ever Married	0.8 (0.55–1.26)	0.7 (0.40–1.41)	0.6 (0.33–1.25)	0.7 (0.34–1.28)
**Socio-economic position**				
Lower	1	1	1	1
Middle	1.7 (0.61–4.92)	1.5 (0.55–4.09)	1.5 (0.54–4.24)	1.4 (0.51–3.72)
Higher	**3.3 (1.27–8.72)**	2.7 (0.99–7.21**)**	2.4 (0.82–6.78)	2.2 (0.82–5.94)
**Religion**				
Christian	1	1	1	1
Muslim	**0.5 (0.35–0.87)**	**0.6 (0.34–0.86)**	**0.6 (0.39–0.91)**	**0.6 (0.40–0.97)**
**HIV/AIDS-related knowledge index**				
Low	1		1	1
High	0.6 (0.28–1.42)		0.5 (0.26–1.15)	0.5 (0.24–1.04)
Comprehensive knowledge	1.0 (0.50–2.14)		0.9 (0.43–1.70)	0.7 (0.40–1.33)
**Risky sexual behaviour index**				
No risk	1		1	1
Some risk	1.6 (0.99–2.49)		**2.1 (1.28–3.53)**	**2.1 (1.31–3.42)**
High risk	**2.9 (1.56–5.27)**		**3.1(1.71–5.51)**	**3.0 (1.68–5.51)**
**HIV/AIDS-related stigma index**				
No stigma	**6.6 (1.93–22.94)**			**3.6 (1.04–12.46)**
Low	**3.7 (1.09–12.31)**			1.9 (0.56–6.67)
Moderate	2.8 (0.72–9.94)			1.6 (0.44–6.12)
High	1			1

Keys: ♣= unadjusted odds ratio, * = only for socio-demographic variables, ** = for socio-demographic variables, knowledge, and risky sexual behaviour, *** = all variables in the model.

**Table 4:** Logistic regression analysis of determinants of VCT utilization among rural men in Ethiopia, 2005

**Variables**	***Ever been tested for HIV***			
	**Univariate OR(95% CI)**♣	Model 1 OR(95% CI)*	Model 2 OR(95% CI)**	Full Model OR(95% CI)***
**Age**				
15–24	**3.9 (1.26–11.73)**	**7.4 (1.87–29.41)**	**6.9 (1.74–27.79)**	**7.4 (1.79–30.75)**
25–34	**7.6 (2.54–22.78)**	**7.2 (2.36–22.02)**	**6.7 (2.21–20.58)**	**6. 8 (2.18–21.11)**
35–44	**4.7 (1.44–15.37)**	**3.7 (1.11–12.39)**	**3.4 (1.03–11.55)**	**3.6 (1.06–12.15)**
45–59	1	1	1	1
**Marital status**				
Never married	1	1	1	1
Ever Married	**1.7 (1.03–2.86)**	**3.0 (1.22–7.23)**	1.7 (0.67–4.47)	2.1 (0.83–5.17)
**Socioeconomic position**				
Lower	1	1	1	1
Middle	**3.5 (1.96–6.43)**	**2.4 (1.33–4.38)**	**2.2 (1.22–3.88)**	**1.9 (1.04–3.54)**
Higher	**8.0 (3.82–16.72)**	**5.0 (2.64–9.46)**	**4.2 (2.22–7.84)**	**3.5 (1.84–6.71)**
**Religion**				
Christian	1	1	1	1
Muslim	1.2 (0.74–2.00)	1.4 (0.85–2.44)	1.6 (0.93–2.70)	1.6 (0.94–2.66)
**HIV/AIDS-related knowledge index**				
Low	1		1	1
High	**2.8 (1.36–5.89)**		**2.3 (1.10–4.97)**	2.0 (0.91–4.23)
Comprehensive knowledge	**3.8 (1.78–8.00)**		**2.8 (1.33–6.00)**	2.1(0.90–4.66)
**Risky sexual behaviour index**				
No risk	1		1	1
Some risk	**2.2 (1.18–4.01)**		2.3 (0.90–5.65)	2.2 (0.89–5.38)
High risk	1.5 (0.66–3.49)		1.8 (0.60–5.35)	1.7 (0.61–4.75)
**HIV/AIDS-related stigma index**				
No stigma	**12.6 (3.90–40.85)**			**9.3 (2.58–33.28)**
Low	**3.4 (1.80–6.39)**			**2.2 (1.09–4.65)**
Moderate	1.6 (0.81–3.11)			1.5 (0.75–3.00)
High	1			1

Keys: ♣= unadjusted odds ratio, * = only for socio-demographic variables, ** = for socio-demographic variables, knowledge, and risky sexual behaviour, *** = all variables in the model.

In model 1, younger age (≤ 44 years), being married and having higher SEP (≥ middle) were associated with greater VCT use than being older, never married and having lower SEP, respectively, in the rural stratum (Table 4). Being Muslim was the only socio-demographic factor that was significantly and negatively associated with VCT utilization in the urban stratum (Table 3).

In model 2, younger age (≤44 years), better SEP (≥ middle), and having better HIV/AIDS-related knowledge (≥ high) were associated with VCT utilization in rural stratum (Table 4). In the urban stratum, only being Muslim and having a history of risky sexual behaviour (≥ some risk) were associated with using VCT (Table 3).

In the full model, stigma was found to be an important predictor of VCT utilization in both urban and rural strata. Age and SEP were found to be positive predictors of VCT utilization in the rural stratum, whereas reporting risky sexual behaviour was a positive predictor in the urban stratum. Urban men who were Muslim were less likely to be tested for HIV than Christians even after adjustment for the more proximal factors (Tables 3 and 4).

## Discussion

This study showed that VCT service utilization among men in Ethiopia was low and the urban–rural difference was striking. Apart from the socio-demographic variables SEP, residence, religion and age; HIV-related stigma and risky sexual behaviour were found to be the most important determinants of VCT utilization among men (aged 15–59 years). The estimated prevalence of ever having used VCT (mainly in urban areas) is comparable with findings from surveys conducted in other SSA countries about 5–10 years ago [12,16,30]. Recently, HIV testing services have been scaled up substantially with an introduction of routine testing i.e. provider-initiated models [31]. Generally, routine HIV testing has been more accepted and it led to substantial increases in the uptake of testing in most SSA countries [31-35]. However, fewer men as compare to women seem to accept the service and have been tested for HIV in areas where this new service model had been scaled up [31].

In this paper, HIV/AIDS-related stigma was found to be strongly and inversely associated with VCT utilization. This is in line with findings from a study conducted in Humara, Ethiopia, and other SSA studies.[8,10,11,16,27,36]. The sociologist Goffman [37] provided the classic definition of stigma as a significantly “discrediting attribute”. He described three different types of stigma: blemishes of individual character, abomination of body, and tribal identity, which refer to traits and/or behaviour that are judged unacceptable in the culture, e.g. an unaccepted sexual practice/behaviour; disfiguring conditions and physical handicaps; and memberships of marginal groups such as sex workers, drug users, migrant workers, and the poor. All these three stigma dimensions exist in relation to HIV/AIDS. AIDS-related stigma may be categorized into two types of reactions to people with HIV: “instrumental AIDS stigma” (originating from a desire to protect oneself from this illness) and “symbolic AIDS stigma”( related to moral judgment) [38], and our stigma index included both these aspects. Unwillingness to care for relatives with AIDS, unwillingness to buy vegetables from vendors with AIDS and not allowing a teacher with AIDS to continue teaching can be categorized as instrumental AIDS stigma. Blaming people with AIDS for bringing AIDS into the community and keeping HIV/AIDS infection secret can be categorized under symbolic AIDS stigma. Such stigma may be due to a perceived association between HIV/AIDS and disliked groups, e.g. commercial sex workers. For example, an HIV-positive person may be stigmatized not only for being infected but also for suppositions relating to his/her sexual lifestyle, sexual orientation and other characteristics e.g. drug use [39]. HIV/AIDS–related stigma has strengthened pre-existing social disparities/inequalities and reinforced the distancing among various groups in a given society, and the strong relationship to sexuality, poverty and gender contributes to this. Moreover, reactions towards people infected with or affected by HIV/AIDS can impede both willingness and ability to adopt HIV preventive behaviour.

Stigma has significant and harmful effects on health and disease transmission by delaying care seeking and failure to disclose health conditions owing to fear of being isolated or rejected, and may result in non-adherence to medical advice[36]. According to Kloos and colleagues, “Stigma and discrimination have taken their toll in Ethiopia not only at the work place, in housing, health facilities, schools, and family and personal relations but also in medical services, discouraging people from being tested for HIV” [4] (p. 8). A review study conducted in South Africa showed that HIV/AIDS-related stigma drives the pandemic out of the public view and is reducing both individual and societal efforts for behavioural change [40]. It is also documented that stigma can lead to violation of human rights which affect the well-being of people living with HIV/AIDS in fundamental ways, such as denial of the right to health care, work, education and freedom of movement. Such adverse effects of stigma can be combated by implementing legal and political reforms [41].

In this study, there were marked differences in HIV/AIDS-related stigmatizing beliefs as well as in the utilization of VCT services between men residing in urban and rural settings. Initially, HIV in Ethiopia was mainly transmitted in urban areas, but it spread to rural areas at a later stage probably through frequent visits of farmers to towns and markets, soldiers’ demobilization, increasing motorized long-distance transportation and resettlement migrations [4,42]. The longer-standing epidemic in urban areas might have given the urban populations more experience in interacting with people living with HIV/AIDS, and over time this may have become a factor in reducing stigma. A study conducted by Hutchinson and colleagues in South Africa also found that low stigma was commonly seen among urban adults with better education and those with higher economic status. The authors mentioned that in rural areas with smaller communities and less anonymity there may be fear about compromised confidentiality, thereby increasing stigmatization from a positive test [12]. A study conducted in the North-Western part of Ethiopia reported that urban dwellers were ten times more willing to give care to their HIV-infected relatives than rural villagers [43]. A similar urban–rural differential has been found in other studies from SSA [11,44]. The difference in VCT utilization between urban and rural areas could probably also be attributed to differences in access to health care facilities offering HIV-testing and HIV/AIDS-related information. In Ethiopia as in other countries in SSA, the distribution of health care facilities is un even and skewed towards urban areas[45].

According to the Ministry of Health report from 2003 [46], high rates of new HIV infections among men in the general Ethiopian population occurred between the ages of 15 and 34 years, and the highest rate was in the 30–34 year age group. Focusing on this age group in the promotion of VCT is therefore likely to be the most cost-effective way of reducing the HIV/AIDS epidemic in the country if we assume that VCT has primary preventive effects [47]. Thus, it is positive that our study found that higher proportions of rural men in this age group had been tested. The higher test proportion found among younger than older rural men was in line with findings from other studies in SSA [11,13,16,48]. The association with younger age that remained after adjustment for education, marital status, knowledge, and sexual behaviour probably reflects the fact that older rural men have a lower self-perceived risk of HIV, but we had not information about this predictor [11].

In agreement with studies conducted in rural Uganda [11,49], and Zambia [16,50] and a cohort study in Zimbabwe [13], the present study demonstrated that VCT utilization among rural men is positively associated with SEP. Other studies conducted in Ethiopia have also shown that having higher educational status, being employed and having better income was associated with ever being tested for HIV [25,26,51]. In our study, the multivariate model indicated that the effect of SEP on VCT use was partially mediated through differences in knowledge, sexual behaviour and stigma since the magnitude of the association had decreased. However, it remained significant in the full model and this could reflect other intermediate factors for which we have not adjusted. Previous studies have found that education is likely to increase awareness and understanding of health-related information as well as confidence in interacting with health care providers [52]. Education also influences knowledge about which types of health care services to use, as well as when and how to use them [53]. It should also be noted that the association between SEP and VCT utilization found in this study could be indicative of differences in freedom to make one’s own health-related choices, and a sense of ability to adopt a particular health protective behaviour. It has been documented that inequalities in SEP result in unequal health outcomes in general [52]. In the same way, variation in use of testing services leads to inequality in access to prevention and treatment of HIV/AIDS.

In contrast to the findings of Yahaya and colleagues in Nigeria [54] the present study found a significant association between religion and VCT utilization among men residing in urban areas, with Muslims being less likely to be tested for HIV. A possible explanation could be higher adherence to religious tenets, which may give protection against the sexual transmission of HIV. In Islam, even though polygamy is allowed for men and divorce is relatively easy, prohibitions against extramarital sex may outweigh the potential risks posed by the former two [55]. Islam also prohibits consumption of alcohol and alcohol is associated with higher likelihood of engaging in risky sexual behaviour [56]. Furthermore, all Muslims should be circumcised [55] and circumcision has been identified as a practice decreasing HIV transmission [57]. Therefore, it is likely that Muslim men have a lower self-perceived risk of HIV and thus are less motivated to be tested for HIV.

As in the findings from a study in Nigeria [58] and another in Ethiopia [59], the present study indicated substantial misconceptions about modes of HIV transmission; e.g. transmission by mosquitoes and through sharing of food. The difference in HIV/AIDS-related knowledge among different subgroups appeared to be associated with VCT utilization, particularly in the rural stratum. However, this association did not remain statistically significant after we adjusted for stigma. Therefore, this indicates that the effect of knowledge on testing primarily worked through an effect on stigma. According to Fisher and colleagues, as cited by Kalichman and colleagues [60], fact-based education about HIV transmission is necessary, but not sufficient for promoting HIV testing.

In Ethiopia as in other SSA countries, heterosexual intercourse is considered as the main route of HIV transmission [46]. The report from the 2000 EDHS showed that only 5% of men used a condom during their last sexual intercourse [61]. In contrast to the findings from some other SSA countries [11,13,49,62], but in agreement with studies conducted in Tanzania [63] and Italy [64], the present study found that risky sexual behaviour was significantly associated with VCT utilization among urban men. One explanation for the significant association could be that urban men who had a history of risky sexual behaviour might have perceived themselves as being at risk of HIV infection and thus be motivated to be tested for HIV. However, risky sexual behaviour history was not significantly associated with VCT utilization in the rural stratum. One possible explanation is that rural men are less educated and may lack adequate HIV/AIDS-related knowledge [11] to realize that such kind of sexual behaviour increases the risk of HIV infection, so they are less motivated to be tested for HIV.

One limitation of this study is the cross-sectional nature of the survey data, which makes it impossible to draw causal conclusions. The non-response percentage was low, and our sensitivity analyses indicated that a higher test experience in this group would not have much affected the overall prevalence estimate for testing. The information in the survey was self-reported, so some degree of under-reporting of socially unacceptable behaviours and attitudes (such as stigma) and over-reporting of socially desirable behaviours are likely. It should also be noted that this study has been based on data collected in 2005, and since then the absolute level of HIV testing experience among men is likely to have changed substantially along with improved access to HIV testing in the country. Another round of the EDHS was conducted in 2010. The data and full report from this survey are not yet available, but these data will also allow us to assess whether predictors of VCT use have changed over time.

As shown in Figure 2, a number of factors that are likely to influence VCT use were not assessed in the EDHS 2005. These include perceived risk of being infected and health-care service factors such as cost, distance, perceived quality, and accessibility. Other studies from SSA have documented that alternative strategies, such as hospital-based, home-based and workplace-based testing, increased access to and uptake of VCT compared to clinic-based VCT [10,65-68]. This indicates that there are a number of facility-related barriers to VCT use. More research is needed to understand these barriers probably, and future research efforts related to this would probably benefit from a mix of quantitative and qualitative methods. However, current evidence indicates that the alternative VCT strategies have the potential to diminish existing socio-economic gradients significantly and alleviate the impact of HIV/AIDS on the most vulnerable households [69].

## Conclusions

VCT utilization among men in Ethiopia is very low. HIV/AIDS-related stigma, area of residence and risky sexual behaviour were major factors affecting VCT utilization among men in the country. Such low utilization of VCT among men in Ethiopia might pose a challenge to the scale-up of HIV prevention efforts. This highlights the need for alternative and improved voluntary HIV testing strategies and alternative models should be tested in terms of acceptability and feasibility in Ethiopian settings.

## Abbreviations

AIDS: Acquired Immune Deficiency Syndrome; CSA: Central Statistical Authority; DHS: Demographic Health Survey; HIV: Human Immunodeficiency Virus; MOH: Ministry of Health; SEP: Socio-economic position; SSA: sub-Saharan Africa; UNAIDS: Joint United Nations Program on HIV/AIDS; VCTSG: The voluntary HIV-1 counseling and testing efficacy study group; WB: The World Bank; WHO: World Health Organization.

## Competing interests

The authors declare that they have no competing interests.

## Authors’ contributions

THL made a substantial contribution to the conception and design of the study, statistical analysis and interpretation of the study findings, and drafting of manuscript. IFS participated in interpretation of the findings and revising the manuscript critically for important intellectual content. KF participated in the conception, design, and interpretation of the study and drafting of the manuscript. All authors read, edited and approved the final manuscript.

## Pre-publication history

The pre-publication history for this paper can be accessed here:

http://www.biomedcentral.com/1471-2458/12/438/prepub
